# Parliamentarizing war: explaining legislative votes on Canadian military deployments

**DOI:** 10.1177/00471178231151904

**Published:** 2023-01-31

**Authors:** Philippe Lagassé, Justin Massie

**Affiliations:** Carleton University; University of Quebec in Montreal

**Keywords:** Canada, legislatures, parliament, war powers

## Abstract

The parliamentarization of military deployments is a burgeoning area of study but has tended to neglect the peculiar cases of legislatures deprived of any war powers. This article contributes to this literature by examining the curious case of Canada. Since Canadian governments are not required to secure parliamentary support to deploy the military, it analyzes why they occasionally have and increasingly do. We propose and test four hypotheses to explain why and when governments willingly choose to involve parliament in war decisions absent constitutional or legal obligation to do so: executive ideology, mission risk, minority parliament, and blame shifting. Our findings suggest that ideology and mission risk have the strongest explanatory and predictive power for when the executive will invite the legislature to vote on a military deployment in Canada. While the desire to avoid blame may contribute to the decision to hold a vote, this is not as influential or statistically relevant. The association between holding a vote and being in a minority parliament, for its part, is negligible and statistically insignificant.

## Introduction

This article examines why parliaments that do not have formal war powers are invited to vote on military deployments by the executive. Recent studies of parliamentary war powers have focused on the normative question of whether legislatures should control military deployments.^1^ Scholars have also examined how legislatures vary with respect to military deployments votes, notably around consistency, effect, and how party politics influence debate and outcomes.^2^ This article contributes to both these literatures by examining decisions by the executive to involve the legislature in military deployment decisions when it is under no legal or constitutional obligation to do so. Given the increased number of expeditionary military operations that many European, North American, and Australasian democracies have undertaken since the end of the Cold War, and the still higher operational tempo that these countries have seen in the two decades since the 9/11 attacks, explaining why executives seek parliamentary support for military deployments when it is not required contributes to our understanding of the politics of military interventions and the influence of domestic politics and institutions on international relations. This research further contributes to wider debates in international relations, notably democratic peace theory,^3^ insofar as the politics and processes involved in deploying militaries shape how democracies approach the use of force. The tendency of executives to hold military deployment votes when they are not required suggests that democratic processes may enable the use of force aboard, not only constrain it.

We propose four hypotheses to explain why executives seek parliamentary support for military deployments when it is not legally or constitutionally required. The first hypothesis holds that votes are tied to the ideology of the party forming the executive. We argue that parties diverge ideologically not only over substantive policy issues but also over procedural matters, including how forces should be committed to international operations. The second hypothesis holds that legislative votes are linked to risks associated with missions. The greater the risk of casualties associated with a military intervention, the more likely the executive will seek democratic legitimacy. The third hypothesis holds that votes are linked with the domestic institutional setting. Minority parliamentary governments are expected to try to secure parliamentary support to prevent legislative backlash over their decision to use force. The fourth hypothesis maintains that the executive initiates these votes to avoid blame for military deployments by attributing the decision to the legislature. Involving the legislature in military deployment decisions can serve to blur the executive’s responsibility or provide political cover when operations lack public support.

These four hypotheses are tested against the case of Canada. Legally, the Canadian executive is not required to obtain the support of the House of Commons to deploy the armed forces, and Canadian governments have regularly deployed the military without consulting the lower house of Parliament. Nonetheless, several Canadian governments have decided to bring such votes before the Commons, either before or after a deployment has taken place. While the practice of holding military deployment votes has elicited some attention in a Canadian context,^4^ the question of why these votes have been held is lacking a comprehensive answer. The Canadian case, moreover, sheds light on other countries where military deployment votes are held, despite the absence of a legal requirement to consult the legislature, as Fonck and Reykers^5^ have examined with respect to the Belgian case. Testing our four hypotheses against the Canadian case therefore promises to advance the study of military deployment votes in states where these decisions formally belong with the executive alone, as well as provide deeper theoretical grounding for the study of these votes in Canada.

Our findings suggest that ideology and mission risk have the strongest explanatory and predictive power for when the executive will invite the legislature to vote on a military deployment in Canada. While the desire to avoid blame may contribute to the decision to hold a vote, this is not as influential or statistically relevant. The association between holding a vote and being in a minority parliament, for its part, is negligible and statistically insignificant.

These findings are relevant to cases beyond Canada. They suggest that the ideological views parties have about the legislature’s role in military deployment decisions matter. Our findings further suggest that combat missions compel the executive to seek legislative support. The importance of combat may therefore explain why legislatures without formal war powers have increasingly been called upon to vote on military deployments since the end of the Cold War and in the aftermath of 9/11. Ideology and combat, furthermore, appear to have been consequential for the increase in military deployment votes in the United Kingdom,^6^ another country where there is no legal requirement to consult the legislature. As a result, the ideology and mission risk hypotheses should inform future research on other cases.

The article begins by reviewing the literature on the parliamentarization of military deployments and presenting our hypotheses for why and when military deployment votes are held in parliaments with no war powers. Next, we outline and compute the Phi Coefficient, a statistical measure we use to evaluate the strength of association between the presence of a vote and each of our four variables as well as to determine statistical significance. Our sample (*n* = 59) consists of Canadian military deployments occurring between 1950 and 2019, of which 15 were put to a legislative vote. Thirdly, we examine how our results align with qualitative analyses of four notable deployment Canadian votes since the end of the Cold War. We find that the explanatory factors that have systematic impact on votes being held since the Second World War are having a conservative party in power and deployments involving combat or the risk of combat. The article concludes with a discussion of the implication of our findings for future research.

## Parliaments and military deployments

Scholarly work on the parliamentarization of military deployments has two strands. The first involves normative studies focused on the democratic imperative to increase legislative involvement regarding the use of armed force, including holding votes.^7^ The desirability of parliamentary involvement mainly rests on arguments for greater democratic legitimacy for decisions that put lives at risk. Conversely, greater parliamentary war powers may undermine military efficiency by slowing down the decision-making process and may limit democratic accountability by blurring the distinction between executive and legislative responsibilities.^8^

The second strand focuses on how legislatures differ in terms of the powers they exercise over military deployments and how political parties have tended to vote.^9^ The increase in military deployments after the end of the Cold War raised interest in studying parliamentary war powers. Mapping the parliamentary control of military missions in 49 democracies between 1989 and 2004, Peters and Wagner^10^ found a trend toward reforming deployment provisions and greater differentiation of war powers between democracies. Examining 21 countries from the Global North from 1990 to 2019, Ostermann and Wagner^11^ notably found that parliamentary votes on military deployments increased over the course of the 1990s and have remained relatively stable into the 21st century. Most deployment votes occurred in Europe, with East Asia and North America respectively accounting for only 10% and 4%.^12^ This suggests that parliaments exert greater influence over deployments in Europe than elsewhere in the world.

Parliamentary war powers refer to the extent to which the legislature controls the use of military force, whether by restraining the scope of executive leeway or by co-deciding.^13^ Roughly a third of democracies around the world have institutionalized parliamentary *ex ante* veto rights, granting legislatures power of prior approval of troop deployments.^14^ The absence of a formal veto right, however, does not mean that parliament has no authority with regards to the use of force.^15^ Some parliaments have the right to be consulted prior to troop deployment; others can debate the decision to send the armed forces after their deployment, or even demand troop withdrawal through *ex post* veto powers.^16^ Parliamentary war powers thus range from two ends of the spectrum: full legislative control – mandatory formal prior parliamentary approval to deploy military forces – on one end, and total executive autonomy – no parliamentary involvement whatsoever – on the other.

While most democracies have some form of parliamentary war powers, a few notable exceptions, such as Belgium and Westminster democracies, have no specific parliamentary control or right to debate the use of military force. Westminster democracies have left the Crown’s historic prerogative power to deploy solely with the executive.^17,18^ Accordingly, decisions on the use of military force belong exclusively with the executive and do not require parliamentary approval or consultation. The United Kingdom recently moved away from this trend by subjecting the executive’s power to a convention of parliamentary approval. Since the 2013 vote on Syria, a convention has emerged such that the British House of Commons must approve major military deployments involving combat.^19^ In contrast, executives in Australia, Canada, and New Zealand have not seen a convention of parliamentary approval develop. Yet, legislative votes on military deployments have nevertheless been held in these states. This thus raises the question this article seeks to answer: Why do executives voluntarily put (some) military deployments to a legislative vote? Otherwise put, why do governments (occasionally) parliamentarize war?

Most studies of the factors that have led legislatures to hold votes have focused on European parliaments equipped with some form of voting powers on military action^20^ and on the legislative determinants of increasing parliamentarization of troop deployments.^21^ A notable exception is Fonck and Reykers,^22^ who focus on executive motivations and strategies for parliamentarization. Analyzing why prior parliamentary approval deployment votes were held in Belgium, where parliament merely has the right to be consulted *ex post facto*, they find that Belgian executives, operating as caretaker governments, drove the decision to hold votes out of risk aversion and to ensure the country’s international credibility. They conclude that future studies should examine other cases of parliamentarization in situations where executives hold exclusive prerogatives to decide upon troop deployments.

In Canada, a typical case of total executive autonomy, the normative debate has been most prominent, with one group of scholars arguing that the democratic principle should see the Commons playing a larger role in military deployment decisions,^23^ and another group defending the executive’s discretion over these matters.^24^ Thus far, studies of why the Canadian House of Commons has irregularly held deployment votes have been limited and comparative in focus.^25^ A comprehensive explanation of why legislative deployment votes are voluntarily held by executives is lacking.

### Hypotheses

The purpose of this analysis is to understand why these votes have been held in Canada and what this case contributes to the study of parliamentary war powers. With much of the cross-national scholarship focusing on European democracies, the Canadian case contributes to furthering the generalizability of research on parliamentary involvement in military action. Since Canadian law does not require the executive to inform, consult or secure the authorization or the support of the House of Commons to deploy the military, even *ex post*, and because Canadian governments have often deployed the armed forces without consulting the elected house of the legislature, there exists a puzzle as to why these votes are occasionally held. Drawing on literature in Canada and internationally, we suggest four hypotheses that may explain why parliamentary deployment votes are held when they are not legally or constitutionally required. These are the executive ideology hypothesis, the mission risk hypothesis, the minority parliament hypothesis, and the blame avoidance hypothesis.

The ideological hypothesis maintains that the choice to hold a military deployment vote is based on philosophical beliefs about how governing authority is exercised in a democracy. As found by Wagner et al.,^26^ European political parties not only systematically differ in their support for military missions, but they further disagree over the role of parliament in deployment decisions. Therefore, greater attention must be paid to the party-political dimension of parliament’s involvement in deployment decisions. We expect parties who believe that the legislature has a duty to check the executive, or that the executive should only make certain significant decisions with the approval of the legislature, will hold military deployment votes, even if not legally bound to. Conversely, parties who believe that the executive should make decisions on its own, with the legislature holding it to account thereafter, will not hold these votes. At the core of this argument is a disagreement about whether democratic representation and deliberation or executive efficiency and accountability should be the primary consideration when military deployment decisions are made. Those who emphasize democratic representation and deliberation hold that decisions involving the use of armed forces should be made by representative legislatures following a proper and thorough debate. Those who emphasize executive efficiency and accountability note that the executive branch is better suited to make decisions about the use of armed forces, given the intelligence and military advice it has at its disposal, and that the democratic propriety of these decisions is ensured by legislatures holding the executive to account for their decisions after the fact.

Examining the cases of France, Germany, Spain, and the United Kingdom, Wagner et al.^27^ found that political parties’ procedural beliefs were generally concomitant with their preferences over substantive policy. Parliamentary involvement was demanded by left-wing parties, sceptical about the use of force, while executive freedom was advocated by right-wing parties, supportive of the use of force. Preferences over substantive policy translate into preferences over parliamentary involvement because increases in transparency and accountability lead to greater opportunities to critique the government’s use of military force.^28^ Ideological preferences toward the use of force follow a curvilinear pattern: support for the use of force is lowest on the far left; it grows as one moves to the center-left, reaches its peak at the center-right, and declines as one moves to the far right.^29^ Left-wing, hard left and hard right parties thus tend to support greater parliamentary control than centrist parties.^30^

**Figure 1.**
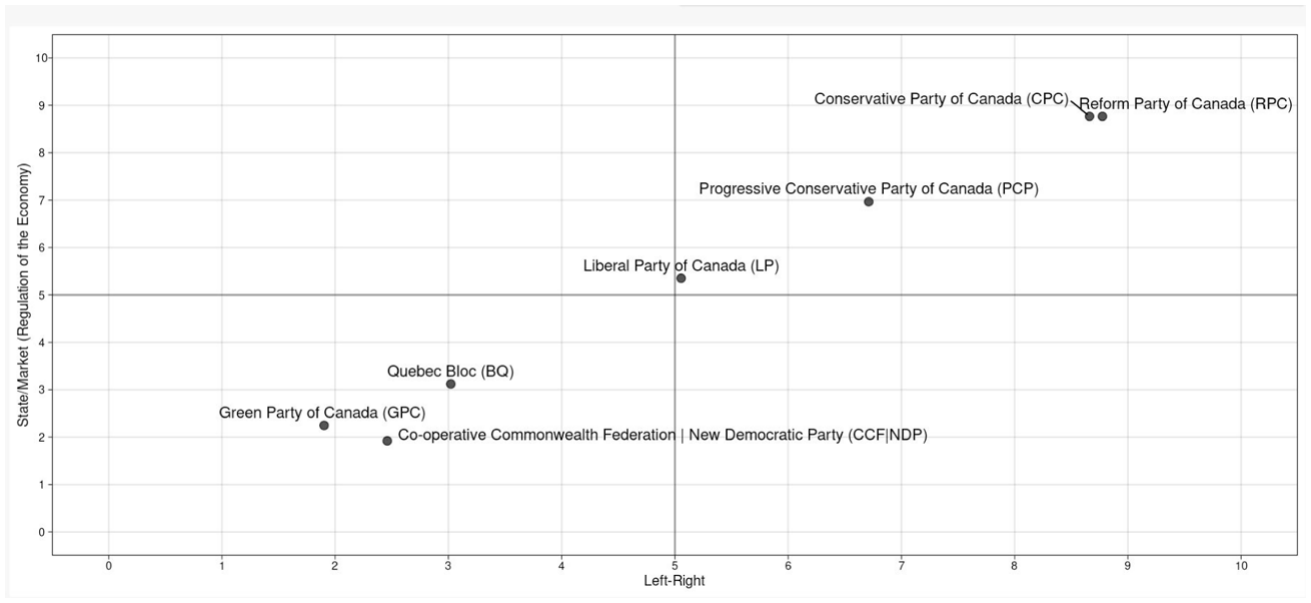
Ideological mapping of major Canadian political parties.

In Canada, only two political parties have ever formed government: the Liberal Party and the (Progressive) Conservative Party. We therefore focus our attention on these two parties alone.^31^ The Liberal Party is a centrist, brokerage party, and has been since the Second World War. The Conservatives, on the other hand, split into two parties in 1987: the right-wing Reform Party (which morphed into the Alliance Party in 2000) and the center-right Progressive Conservative Party. Both parties reunited in 2003 under the right-wing Conservative Party. Figure 1 maps the ideological positions of major Canadian parties based on the Parliament and Government Composition Database (ParlGov), which aggregates party positions on three dimensions on a 0–10 scale: left/right, state/market, and liberty/authority.^32,33^ Data is based on party expert surveys and the Comparative Manifesto Research Project, which is based on content analyses of election programs.^34^ The Liberal Party scores close to the center on all three dimensions: left/right (5.06), state/market (5.35), and liberty/authority (3.46). The Progressive Conservative Party scores closer to the center-right (6.71, 6.97, and 5.99), whereas the Reform (8.77, 8.77, and 9.07) and the Conservative party (8.66, 8.77, and 9.07) are right-of-center on all three dimensions of political ideology. The results are consistent with Cross and Young’s^35^ survey of Canadian political party members,^36^ as well as Canadian party embeddings based on millions of speeches at the House of Commons from 1901 to 2018.^37^

As Canada’s ‘natural governing party’, the Liberals have tended to favor executive decision-making. While the Liberals voiced support for greater parliamentary control when pursuing autonomy from the United Kingdom prior to the Second World War, and during the few periods when they have been in the opposition, empowering the legislature has not been a core part of their ideology or party preferences. The Conservatives, by contrast, have voiced stronger support for legislative checks on the executive, owing to their longer periods in opposition. Of note, the Reform Party advocated for more direct democracy and a rebalancing of executive-legislative relations in Parliament’s favor, including requiring the government to consult the Commons prior to deploying armed forces.^38^ Indeed, when the Progressive Conservatives were in power from 1984 to 1993, the government consulted the Commons on military deployments. When the new Conservative Party reunited in 2003, it retained this ideological position in favor of greater parliamentary control of executive decision-making, notably with respect to military deployments.

Considering these divergent ideological beliefs between Canada’s two governing parties, we make the following hypothesis:

- *H1 (Executive Ideology): Votes will be held when the (Progressive) Conservative Party of Canada is in power.*

The second hypothesis focuses on the risks associated with certain types of military deployments. According to the mission risk hypothesis, the likelihood that a legislative vote will be held increases as the dangers surrounding a deployment become more pronounced.^39^ Indeed, war is a particularly risky endeavor for democratic executives facing accountable institutions and electoral competition.^40^ As they contemplate the possibility of putting their citizens’ life at risk, democratic executives may fear *ex post* punishment and thus avoid policies that increase the chances of electoral defeat or leadership change.^41^ Political leaders must also contend with militaries that are reluctant to deploy on high risk missions, which increases the need to build legitimacy for these operations.^42^ Building on this assumption, several studies have demonstrated that democracies with greater legislative war powers are generally less prone to take part in military interventions involving combat operations.^43^ Furthermore, legislatures are more likely to have a role in making decisions that involve casualties since these decisions tend to be controversial and costly for executives. As Peters and Wagner^44^ found, the experience of losing wars with a high number of casualties increases the probability of parliamentary control.

Opposition parties and executives differ in motivations to involve parliament in risky military decisions. Opposition parties are expected to call upon the legislature to exercise its representative function because dangerous deployments tend to attract greater attention. Securing parliamentary involvement will thus increase their visibility and influence over salient deployments.^45^ Executives, for their part, are expected to voluntarily relinquish their authority over the use of force out of concerns for the credibility and legitimacy of the military mission.^46^ Legitimacy derives from holding to account the executives responsible for the decision to put the lives of citizens at risk and from involving those mostly affected in the decision-making.^47^ In these circumstances, governments will often aim to garner a broad consensus from opposition parties to support their decision to put their citizens’ lives at risk.^48^ Furthermore, securing a parliamentary mandate signals resolve and democratic support to put troops into harm’s way for political goals, while increasing the credibility of the international commitment.^49^ Democratic executives may thus choose to hold a vote on a high-risk mission to demonstrate resolve to a potent adversary, in contrast with low-risk deployments where there is no such need.

We rely on a conventional proxy to measure the risks tied to a military deployment: whether the mission involves combat or not. Combat operations expose both armed forces and civilians to a higher risk of casualties.^50^ Canada’s military doctrine states that combat operations are those ‘where the use or threatened use of force is essential to accomplish a mission’. Non-combat operations are those where weapons may be present, but ‘their purpose is primarily for self-defence’.^51^ We rely on the rules of engagement (ROE) set by the Canadian military to ascertain the offensive or defensive mandate to use armed force in each deployment.^52^ This leads us to make the following hypothesis:

- *H2 (Mission Risk): Votes will be held when a deployment involves combat.*

The third hypothesis maintains that votes are likelier when no party holds a majority of legislative seats,^53^ given that legislative constraints significantly increase for executives operating with a minority parliament. The logic of this hypothesis rests with the institutional constraint argument of the democratic peace,^54^ which contends that executive autonomy diminishes as the number of institutional parties able and willing to veto executive action increases. As parliamentary executives need the support of parliament to make policy, the fewer legislative seats they hold, the more constrained are their policy actions.^55^ This is even the case of executives endowed with total autonomy regarding the use of force, for they nevertheless require support from parliament to fund the armed forces, whether through annual or incremental budget allocations.^56^

Minority parliamentary governments face greater accountability to the legislature, with party discipline insufficient to prevent their decisions being overturned by a majority of parliamentarians. In turn, opposition parties are likelier to challenge the executive on foreign policy issues.^57^ Scholars have thus generally found that domestically weak executives are more risk averse with regards to using military force abroad.^58,59^ They are more likely to avoid policies that will invite legislative challenge and possibly force an election. To overcome their legislative weakness, minority parliamentary governments are expected to try to secure parliamentary support for military deployments. We thus hypothesize the following:

- *H3 (Minority Parliament): Votes will be held during minority parliaments.*

The fourth hypothesis focuses on executive efforts to avoid blame for unpopular military deployments by shifting blame toward the legislature. Blame shifting involves the executive seeking to involve the legislature in decisions to blur its responsibility and accountability. In contrast with mission risk, it is not the fear of putting lives at risk that drives the executive’s desire to hold a legislative vote, but the fear of electoral punishment for making an unpopular decision, irrespective of deployment type. As Weaver^60^ argued, political leaders have a strong incentive to avoid blame for potential unpopular decisions. Avoiding blame, in fact, is a more powerful motivator than taking credit, since voters are more likely to notice failures than reward accomplishments. The underlying motivators for blame avoidance rest in leaders’ risk aversion and the desire to minimize electoral costs.^61^ Anticipating a blameworthy event, officeholders will seek to shield themselves from censure, notably by delegating responsibility to others, diffusing blame to as many policymakers as possible, and ‘passing the buck’ to other institutional bodies.^62^

Blame avoidance behavior has mostly been studied in domestic politics.^63^ Focusing on international affairs, Lindsay^64^ observed that this is an important aspect of how the United States Congress engages in foreign policy questions. Lagassé^65^ argued a similar point when he described Canadian military deployment votes as a form of ‘laundering’, whereby the executive seeks to confuse voters about responsibility for the decision by making it a legislative matter. Examining executive-driven parliamentarization in Belgium, Fonck and Reykers^66^ further emphasized how seeking prior parliamentary backing may serve the interest of executives by deflecting blame toward parliamentarians. In short, governing parties may seek to share the burden of responsibility for controversial military deployments with parliament by securing a broad legislative mandate with the support of opposition parties.

This logic resonates with the argument that elite consensus inoculates governing parties from electoral punishment and enables executives to defy public opposition.^67^ When opposition parties vote with the government, they express confidence in the government and support for its policy. They thus become poorly placed to hold the executive to account^68^ and tend to be muted in their critiques.^69^ Opposition parties are, furthermore, likely to vote against unpopular deployments given their incentive to mobilize public dissent to their political advantage.^70^ Nevertheless, majority governments in parliamentary systems are unlikely to fear dissension by legislators given their hold on a majority of legislative seats and strict party discipline.^71^ Minority governments facing an unpopular intervention choice are, on the other hand, unlikely to deploy troops in the first place.

Blame avoidance is tied to how unpopular a deployment decision is with the public. The greater the public disagreement with a decision to use force, the more likely governing parties will seek to blur their accountability and responsibility, and therefore be incentivized to secure a parliamentary mandate that confuses voters about where the decision to deploy was made. If, however, a mission is not unpopular and hence unlikely to cause electoral backlash, the executive will have fewer incentive to put military deployments to a vote. We thus propose the following hypothesis:

- *H4 (Blame Avoidance): Votes will be held when the deployment is unpopular.*

To measure public opinion, we rely on a simple majority of respondents expressing opposition to a military deployment in a poll. We use the repository of polling data of ODESI (Ontario Data Documentation, Extraction Service and Infrastructure) to identify the best polling question and timing. When available, we use opinion polls asking respondents about their general support to Canada’s military intervention prior to the deployment decision. This choice of timing best captures the polling environment in which the government was at the time of its decision to seek, or not, a parliamentary mandate. In the absence of such polling data, we rely on the closest available poll. We further indicate when general attitudes shifted from opposition to support, and vice-versa, after the deployment decision to account for potential rally-round-the-flag effects.^72^ Such situations lead to less need to avoid blame.

## Data and method

The pattern of deployment votes is expected to result from a multicausal interplay of the four hypothesized explanatory factors developed above. Each driver – executive ideology, mission risk, minority parliament, and blame avoidance – may lead individually or in combination to a deployment vote. To assess the relative explanatory power of each, we examine Canada’s military deployments from 1950 to 2019. The data collected and presented includes overseas deployments of 50 military personnel or more, excluding undisclosed special operations missions, military exercises, and naval operations. Data was collected from *Hansard*, the official record of the Canadian House of Commons, and from a study produced by the Library of Parliament.^73^

Given the size and character of our sample, we use cross-tabulation for a simple bivariate analysis, the results of which are summarized in Table 1. The strength of the four hypothesized relationships between voting and executive ideology, mission risk, minority parliament, and public opinion respectively is captured by the Phi Coefficient. As such, it is appropriate for the study of dichotomous variables, meaning that each only has two possible values. Our data satisfy this: executive ideology is coded as either rightist (conservative) or centrist (liberal), mission-risk as either combat or non-combat ROE, minority parliament as either present or not, and blame avoidance is expressed in terms of public support or opposition. The outcome is also binary in that a vote is either held or not. The Phi Coefficient is derived from Chi-square, a statistic commonly used to test relationships between categorical variables. From an interpretive perspective, the Phi Coefficient differs from Chi-square in that the results are limited to a range of −1.0 to 1.0, similarly to the Pearson Correlation Coefficient used in linear regression. Assessing the strength of the association is thus straightforward.

**Table 1.** Analytical results.

Phi coefficient			
Executive ideology	Mission risk	Minority parliament	Blame avoidance
0.3658	0.4636	0.1885	0.2550
0.005**	0.000***	0.148	0.050*

* *p* ≤ 0.05.

** *p* ≤ 0.01.

*** *p* ≤ 0.001.

Using the Phi Coefficient as a formal statistical method requires a null hypothesis (H0). Ours holds that there is no association between the presence of a vote and executive ideology, mission risk, minority parliament, and blame avoidance, respectively. The following section presents the analytical results.

## Patterns of military deployment votes

**Figure 2.**
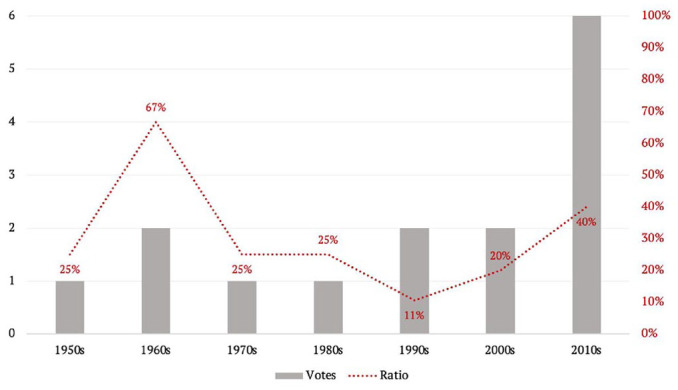
Deployment votes in parliament.

Among the 59 Canadian military deployments that occurred since the Second World War, only a quarter were put to a legislative vote (for details, see the Appendix). These votes were held infrequently until the last decade, which was marked by a surge in occurrence (see Figure 2). The surge in votes is not the result of a greater number of deployments during the 2010s. In fact, Canadian military deployments increased sharply in the 1990s with a record of 19 interventions, following a relatively constant pace of three-four deployments per decade before then. The 2000s and 2010s maintained a high pace, with 10 and 15 deployments respectively. As a share of deployments, however, the 2010s witnessed the greatest number of votes since the 1990s.

Consistent with our first hypothesis, a cursory view of the data shows that close to half of all deployment votes (11 out of 15, or 73%) were held under (Progressive) Conservative governments (see Table 2). While Liberal governments also held votes, these represented exceptions to the trend. Out of the 34 deployments undertaken by these governments, only four (12%) were put to a vote. From this, it seems fair to conclude that deployment votes are more likely to be held under a Conservative government. In this case, the Phi Coefficient is 0.3658, suggesting a weak to moderate positive association between holding a vote and executive ideology. The results are statistically significant at the 0.005 level as denoted by the *p*-value. This is lower than most thresholds for statistical significance, meaning that the probability of our results occurring due to chance is close to none.

**Table 2.** Executive ideology.

Vote	Government		Total
	Conservative	Liberal	
No vote	14	30	44
Vote	11	4	15
Total	25	34	59

Pearson χ^2^(1) = 7.8957.

Φ = Cohen’s *w* = fourfold point correlation = 0.3658.

Φ^2^ = 0.1338.

*p* = 0.005.

The inconsistent cases are the 14 deployments that were not subject to a vote held under (Progressive) Conservative governments, as well as the single deployment votes held by the St-Laurent, Pearson, P. Trudeau, and J. Trudeau governments. Limited sample size prevented us from testing Progressive Conservative and Conservative governments separately. Descriptive data, however, point to a clear difference: Progressive Conservative governments put fewer deployments to a vote – 50% of deployments under Diefenbaker and 25% under Mulroney – than the Conservative Harper government, which held a vote on 64% of its deployments. With regards to the Liberals, both the Chrétien and Martin governments refrained from putting any of their deployments to a vote, in contrast with the St-Laurent, Pearson, P. Trudeau, and J. Trudeau governments, having each put a single deployment to a vote.

We conduct similar tests for our remaining hypotheses. With regards to mission risk, 60% of Canada’s combat operations were put to a legislative vote (see Table 3). Conversely, only six out of 44 non-combat operations (or 14%) were put to a vote. The latter thus represent exceptions to the overall trend: combat operations seem more likely than non-combat operations to be subject to a legislative vote. With a Phi of 0.4636, we find a moderate positive association. The results are significant at a *p*-value of 0.000. Hence, whether an operation does or does not involve combat matters. However, the mission risk hypothesis faces three important inconsistent cases: the wars in Korea, Kosovo, and Afghanistan (2001–2005). These deployments – and more importantly the war in Korea, which resulted in 516 Canadian casualties – undermine the argument that combat is a sufficient condition for a vote.^74^

**Table 3.** Mission risk.

Vote	Combat		Total
	Combat	Non-combat	
No vote	6	38	44
Vote	9	6	15
Total	15	44	59

Pearson χ^2^(1) = 12.6826.

Φ = Cohen’s *w* = fourfold point correlation = 0.4636.

Φ^2^ = 0.2150.

*p* = 0.000.

Our third hypothesis is not confirmed as we do not find convincing evidence for the relationship between minority parliaments and deployment votes. The observed Phi of 0.1885 suggests no to negligible association and the *p*-value ⩾0.1 means that the results are not significant enough for us to reject the null hypothesis (H0). Most deployments (58%) undertaken by a minority government were not put to a vote in the House of Commons (see Table 4). Furthermore, more than a fifth of the deployment votes were held by majority governments. That being said, only 12 of the 59 military deployments (about 20%) were decided by minority governments. These governments therefore thus seem more prudent toward the use of force; but when they do deploy the military, they are only slightly more likely to seek parliamentary support than majority governments.

**Table 4.** Minority parliament.

Vote	Parliament		Total
	Minority	Majority	
No vote	7	37	44
Vote	5	10	15
Total	12	47	59

Pearson χ^2^(1) = 2.0969.

Φ = Cohen’s *w* = fourfold point correlation = 0.1885.

Φ^2^ = 0.0355.

*p* = 0.148.

Finally, a Phi of 0.2550 suggest a weak, positive relationship between holding a vote and avoiding blame. This is significant at the 0.05 level, the conventional, borderline threshold denoting significance. While weak, we may still reject the null hypothesis (H0) in favor of our fourth hypothesis (H4) that blame avoidance matters in the executive decision to hold a deployment vote. Out of the three hypotheses where a degree of association is confirmed, this is the weakest one. Twice as many popular than unpopular deployments were put to a legislative vote (see Table 5). This trend further holds for situations were majority opinion switched following the deployment decision. Moreover, roughly one out of five deployments that enjoyed public backing were nevertheless subjected to a vote in parliament. Overall, Canadians supported many more (83%) military deployments than they opposed (17%).

**Table 5.** Blame avoidance.

Vote	Public		Total
	Oppose	Support	
No vote	5	39	44
Vote	5	10	15
Total	10	49	59

Pearson χ^2^(1) = 3.8357.

Φ = Cohen’s *w* = fourfold point correlation = 0.2550.

Φ^2^ = 0.0650.

*p* = 0.050.

Overall, mission risk holds slightly greater individual predictive force of deployment votes than executive ideology. Both are stronger factors than blame avoidance, while minority status does not have any meaningful effect. With regards to combat operations, six were not put to a vote by Liberal governments: the wars in Korea, Kosovo, and Afghanistan (2001–2005). This contrasts with the Mulroney and Harper governments, which subjected every combat deployment they undertook to a parliamentary vote, irrespective of their parliamentary status or public support. Although the Mulroney’s Progressive Conservatives were seen are more centrist than Harper’s Conservatives, they were ideologically closer than with the Liberals on this issue. In fact, a clear systematic pattern consists in every Progressive Conservative and Conservative governments since the Second World war holding votes on combat operations. The Mulroney government put two combat deployments (the Persian Gulf war and the military intervention in Somalia) to a vote, in addition to a UN peace operation in Iran-Iraq, while the Harper government systematically held votes for combat operations, whether it held a minority or majority of parliamentary seats in the House of Commons. A combination of executive ideology and mission risk thus seem to represent sufficient conditions for Canada’s deployment voting behavior.

Most importantly, combat appears to be the determining factor behind deployment votes since 2006, irrespective of other conditions. Since that time, combat missions have been systematically brought to the Commons for a vote, whether by the Harper or the J. Trudeau governments, during minority and majority parliaments, and with or without public support.^75^ The presence of a Conservative government may thus no longer be a necessary condition, given the Trudeau government’s 2016 Iraq vote. However, it is too soon to say whether the practice of holding combat deployment votes that began under the Mulroney government and was systematically maintained by the Harper and J. Trudeau governments will become a constitutional convention.

Another pattern is noteworthy. The Chrétien, Martin and J. Trudeau Liberal governments followed our expectations with regards to the diminishing appeal of blame avoidance when interventions had public support. Indeed, when considering two changes in public support post-decision – on NATO’s Implementation Force in the former Yugoslavia in 1995 and the non-combat operation against the Islamic State in 2016 – every deployment undertaken by these governments enjoyed positive public backing. It could be that their deployment choices were governed by public support. Conversely, these governments did not deploy troops on unpopular missions. This is in sharp contrast with the Harper government, which saw several of its deployment decisions face public disapproval, which lends credence to the idea that the Conservatives used votes to avoid blame or launder their decisions through the legislature.

In sum, the pattern of deployment votes illustrates a growing parliamentarization of war, whether in terms of the mounting number of deployments put to legislative vote, the importance of government ideology in accounting for seeking to secure parliamentary support, the recent tendency to hold votes on combat deployments, and the influence of public support on recent Liberal governments.

## To hold or not to hold a vote

To better disentangle the motivations leading governments to hold a deployment vote, we examine four noteworthy post-Cold War cases in greater detail: Brian Mulroney’s remarkable decision to put participation in the Persian Gulf War to a vote; Jean Chrétien’s and Paul Martin’s refusals to seek parliamentary approval; Stephen Harper’s decisions to put Canada’s combat deployments in Afghanistan to a vote; and Justin Trudeau’s decision to hold a vote on ending combat operations against the Islamic State. This description of the events and politics surrounding the votes serves to highlight how our four hypotheses operated, or not, since the end of the Cold War.

### Mulroney’s precedent

In January 1991, the Progressive Conservative government of Brian Mulroney was the first since 1939 to hold a parliamentary vote on Canada’s participation in a war overseas. Although the Conservatives held a majority in the Commons, Mulroney’s decision to hold a vote on Canada’s involvement in the Persian Gulf War reflected a view within his party that the decision to send armed forces into harm’s way should be made with the support of the Commons.^76^ The Commons held extensive debates leading up to the Persian Gulf War, which saw the Liberal official opposition arguing strongly against the use of force by the Canadian military but ultimately voting in favor.

According to Kim Richard Nossal,^77^ two factors were pivotal in leading the Mulroney government to hold the debates and vote: the belief that parliamentarians should be consulted, and the significance of the proposed use of force against the Iraqi regime to liberate Kuwait. This perspective echoes our ideology and mission risk hypotheses. The Mulroney government opted to involve Parliament more in foreign policy debates by seeking prior legislative approval of Canada’s participation in two combat operations: the Persian Gulf War and the U.S.-led United Task Force in Somalia. In explaining his choice to consult the Commons, Mulroney^78^ stated in his memoirs that, given the risky military operations in the Middle East, he ‘wanted the representatives of the people to have a final say before a possible war began’. This reflects Conservative ideology about including the legislature in making military deployment decisions, however *pro forma*, a position that would not only endure after the Progressive Conservative split but become stronger when the party was reunited.

Mulroney’s parliamentarization of Canadian military deployments is also seemingly consistent with blame avoidance. In seeking parliamentary approval to commit Canada to the Gulf War, Mulroney was trying to legitimize decisions he had already made in the face of significant popular opposition.^79^ When Parliament was recalled on 15 January 1991 to reaffirm ‘its support of the United Nations in ending the aggression by Iraq against Kuwait’, the Mulroney government had already authorized Canada’s CF-18s to conduct ‘sweep and escort’ missions for American aircraft bombing Iraqi forces.^80^ Yet polls indicated at best ambivalent support to Canada’s participation in the Gulf war and considerable dissatisfaction with the Mulroney government.^81^ Some Tory advisers were particularly concerned that involvement in a protracted war in the Middle East could result in electoral punishment, especially in Quebec, where was the government’s largest electoral base and the greatest public dissent.^82^ In a Gallup poll conducted in December 1990, an overwhelming majority (70%) of Quebeckers expressed their opposition to Canadian war participation, above the average of 51% of support outside Quebec.^83^ While a rally effect took place after the beginning of the war, with a sudden rise in public support in mid-January, it was short-lived and had fell by February.^84^ Seeking parliamentary support could therefore be seen as a means of shielding the government from blame in the face of public opposition.

Although he did not need it to win the vote, the Mulroney government could not have reasonably expected the support of opposition parties. The Liberals and the NDP had consistently opposed the government’s military policy and voted against the government’s motion in November to support the United Nations Security Council approving ‘all necessary means’ against Iraq should it fail to withdraw from Kuwait by mid-January.^85^ At the beginning of the parliamentary debate in January, newly elected Liberal leader Jean Chrétien tabled an amendment calling ‘to exclude offensive military action by Canada’ and urged Canadian troops to be called back if war erupted.^86^ Chrétien’s stance and leadership were attacked by former Liberal Prime Minister John Turner, who rose in Parliament in favor of the Progressive Government’s motion to reaffirm Canada’s support to the United Nations’ actions against Iraq’s aggression. Perhaps as a result of Turner’s intervention, or because war had broken out in the middle of the parliamentary debate, the House overwhelmingly approved the government’s motion by a vote of 217 to 47, with almost every Liberal and Bloc Québécois MP voting in favor.^87^ Mulroney thus insulated his government from a degree of blame. As Davis argued:

The strong support in the House for the government’s resolution helped portray the decision to participate in Operation Desert Storm as non-partisan and allowed the government to avoid potentially embarrassing questions within the House of Commons during Question Period. It might also have allowed the government to escape blame, or at least share it with all those who supported the war effort, if Canadian troops were killed or injured in the war.^88^

### Liberal preference for executive autonomy

Despite this success, the democratization of Canadian foreign policy was specific to the Mulroney government, as his two Liberal successors, Jean Chrétien and Paul Martin, put an end to seeking parliamentary approbation altogether. Both eschewed military deployment votes during their premierships from 1993 to 2006. Chrétien and Martin held take-note debates for some operations, but their governments saw no need to hold votes or secure the Commons’ approval.^89^ Most notably, even the 1999 Kosovo airstrike campaign and combat operations in Afghanistan in 2001–2005 were not deemed worthy of parliamentary approval. In particular, the 2005 counterinsurgency operation in Kandahar was a dangerous combat mission that would likely see the Canadian Armed Forces (CAF) engaged in combat and taking casualties.^90^ Nonetheless, following the practice of the Chrétien government, the Martin Liberals deployed the CAF to Kandahar without a holding a vote, despite not having a majority in the Commons.

The Chrétien and Martin Liberals were comfortable with the notion that governments govern and Parliaments hold the government to account for their decisions, including going to war.^91^ They failed to even respond to the recommendation of the Senate Standing Committee on Foreign Affairs that Parliament approve military interventions (SSCFA 2000). Both Liberal governments were repeatedly criticized by the opposition for committing Canada to war without proper parliamentary consultation. Liberal members defeated every motion tabled in the House to require a parliamentary vote before deploying the Canadian military abroad, arguing that this practice would unduly constrain the executive.^92^

### Harper’s parliamentarization of combat operations

The ideological principle that the Commons should be consulted on military deployments resurfaced when the Conservative Party of Canada led by Stephen Harper was formed in 2003. The issue of the Commons’ role in military deployments was particularly salient at the time, as Canada’s involvement in Afghanistan deepened. In their 2005–2006 electoral platform, the Conservatives promised to ‘Make Parliament responsible for exercising oversight over. . .the commitment of Canadian Forces to foreign operations’.^93^ Involving the House in military deployment decisions was one of Harper’s first acts as prime minister following the 2006 election, which saw the Conservatives defeat the Martin Liberals. In May 2006, the Conservative government held a vote in the Commons to extend the Kandahar mission by 2 years. With only a plurality of seats, Harper was aware that his government might lose the vote. Therefore, he made clear that he would extend the mission by 1 year regardless of the result.^94^ But the act of holding the vote, however symbolic, was important. The vote would help establishing a precedent for consulting the Commons on military deployments and would allow the Conservatives to fulfill an electoral pledge and long-standing ideological goal.^95^

Harper’s decision to bring the Kandahar extension to the Commons was also driven by blame avoidance. The Conservatives recognized that public opposition to the mission would increase if the military took significant casualties.^96^ Against this backdrop, the Conservatives were aware that, since the Liberals had initiated the Kandahar mission, many of their MPs would be loath to vote against it; indeed, there were several Liberals who believed in the deployment and Canada’s efforts in Afghanistan. Securing bipartisan parliamentary approval could thus insulate the minority Conservative government from blame if public opposition grew. Harper Conservatives thus actively sought the support of opposition parties^97^ and it paid off. The Kandahar extension vote was held on 17 May 2006 and passed 149 to 145, thanks to 30 Liberals who voted with the Conservatives. Not only were the Liberals divided, but the Commons had also approved of his mission extension, and the principle of the House voting on military deployments was revived after more than a decade of Liberal governments refusing to hold votes.

The Harper Conservatives held a second vote on extending the Kandahar operation in 2008. Even more than the 2006 vote, the blame avoidance appeal of doing so in a minority parliament was palpable. Under the leadership of Stéphane Dion, the Liberals had begun to call for the end of Canada’s combat mission in the face of accumulating casualties and growing public dissent. This presented a risk for the Harper Conservatives; they alone could be blamed for an increasingly unpopular mission. Avoiding blame and blurring responsibility for the mission was therefore an important goal for the Conservatives. As part of this effort, the Harper Conservatives first set up a bipartisan panel headed by former Liberal cabinet minister John Manley to advise them on an extension of the mission. The Manley Panel ultimately recommended that the Kandahar mission be extended indefinitely, provided that certain capabilities and conditions were met. Armed with this endorsement, Harper entered into negotiations with Dion on an extension. The two leaders agreed that the mission would be extended, but only to 2011. Once this agreement had been reached, a vote was held in the Commons, with both the Conservatives and Liberals supporting the motion.^98^ Having both major parties vote in favor of the extension ensured that the mission was taken off the agenda as a significant issue during the 2008 election that followed shortly after the vote was held.

Consensus between the Conservatives and Liberals shaped how the Harper government approached Canada’s next deployment to Afghanistan as the Kandahar mission was coming to an end. In 2010, while still governing with a minority, the Harper Conservatives announced that the CAF would begin a new peace enforcement operation in Afghanistan’s capital. Unlike the Kandahar deployment, the 2011 Kabul mission was not expected to involve combat and the risks of casualties was far lower. Owing to this lower risk and non-combat objectives, the Liberals agreed with the Harper Conservatives that a vote was not necessary for this deployment.^99^ This episode undermines the minority parliament and blame avoidance hypotheses, for neither the Conservatives nor the Liberals were inclined to hold a vote despite a majority of Canadians opposing continued military involvement.^100^ While executive ideology certainly mattered, Conservatives were coy on why they chose not to put this foreign deployment decision to a vote.^101^ Given its consistency with the fact that no other non-combat deployments were put to a vote, it is fair to conclude that Conservatives’ ideological preference for parliamentary approval only extended to combat operations.

### Trudeau cementing a pattern

During the October 2015 federal election, the Liberals under the leadership of Justin Trudeau were elected with a majority of seats in the House of Commons. During the election, the Liberals pledged that they would end the CF-18 airstrike operations in Iraq and Syria, while strengthening the advising and assisting mission and increasing Canada’s other efforts to address the impact of the Islamic State in the region. Unlike the Chrétien and Martin governments, and notwithstanding their majority in the Commons, the Trudeau Liberals brought this adaptation of the mission before the Commons for a vote in March 2016. This decision is best explained by mission risk.

The Trudeau Liberals felt that the combat votes held under the Harper government established a new practice that they should follow. Indeed, despite its non-combat ROE, the advising and assisting mission involved significant risks and might result in additional casualties under their government.^102^ Canadian troops would operate on the frontlines of the war to support Kurdish forces in their offensive operations against ISIS insurgents in Iraq, including by targeting and coordinating attacks.^103^ Despite having a majority, therefore, the previous decade of holding votes for combat missions convinced the Trudeau Liberals that a vote was prudent. By contrast, the Trudeau government has not held votes when they deployed the CAF to non-combat missions in Latvia, Ukraine, or Mali. While the Latvia and Mali missions involved risks, they did not involve combat and hence were judged not to require a vote. Although they had deployed the CAF on non-combat operations without a vote during the Harper government, the Conservatives nonetheless called on Liberals to hold a vote on the Mali deployment.^104^ The Liberals ignored the opposition’s demands, thereby further ceiling the new trend of voting only on combat operations.

Trudeau’s decision to hold a parliamentary vote could also have been motivated by blame avoidance. In contrast with other military commitments under its premiership, Prime Minister Trudeau faced public opposition against his decision to end to Canada’s airstrikes against the Islamic State.^105^ However, there is no available evidence to support this motivation. On the contrary, officials familiar with the decision raised the fact that holding a vote helped garner legitimacy when putting soldiers’ lives at risk, and that there was little hesitation to holding a vote, ‘especially since the previous government had done the same’.^106^

In sum, these four sub-cases highlight the setting of a new trend whereby legislative approval is increasingly sought for risky military deployments. What began as a precedent under the Conservative Mulroney government, then ignored by his the Liberal two successors, was implemented systematically by the Harper Conservatives and was maintained by the Trudeau Liberals. Blame avoidance also proved a potent explanation of two deployment votes under the Harper government, and consistent with Mulroney and Trudeau’s decisions to hold votes on unpopular deployments. Minority parliament, however, did not prove an important factor, further lending credence to the results of our statistical analysis.

## Conclusion

This article has argued that ideology and mission risk explain Canadian military deployment votes better than the institutional dynamics of a minority parliament or blame avoidance by the government. Since 2006, moreover, the mission risk hypothesis has provided greater explanatory power and is arguably a strong predictor of when the Canadian House of Commons will be asked to approve a military deployment in the future.

Our findings with respect to the Canadian case contribute to the wider literature on the parliamentarization of military deployments in two ways. First, the importance of ideological factors in the Canadian case reinforces the impact of party politics on deployment votes and decisions.^107^ The Canadian case, moreover, suggests that a connection may exist between conservative, right-of-center parties and deployment votes in Anglo-American countries. This finding is counterintuitive, since opposition to deployments has been associated with the political left, which would suggest that left-wing parties would be more inclined to demand a legislative check. It is noteworthy, however, that Labour Prime Minister Tony Blair was reluctant to hold a vote on the 2003 Iraq War in the United Kingdom, and that subsequent military deployment votes in the United Kingdom were held during the Conservative governments of David Cameron. Conservative Prime Minister Theresa May, however, substantially weakened the expectation that the executive would consult the House of Commons, which suggests that the conservative parties still wish to retain executive discretion.^108^ Further complexifying this discussion is the Australian case, where the conservative Liberal Party government of John Howard reluctantly held votes on Australia’s involvement in the Afghanistan and Iraq, while resolutely defending the executive’s discretion, as did Liberal Prime Minister Tony Abbott.^109^ Future research is needed to determine examine whether conservative government are indeed more likely to hold votes in Westminster states.

Secondly, our findings highlight the significance of mission risk for legislative involvement in deployment decision and suggest that this factor should be a differentiator when studying deployment votes. While studies have noted that combat reduces the likelihood that legislatures with established war powers will approve a deployment,^110^ mission risk could also influence whether parliaments who lack formal war powers will hold deployment votes or not. Indeed, the increase in combat operations since the end of the Cold War and after 9/11 could explain why there has been greater legislative involvement in military deployment decisions in parliamentary democracies. Combat missions, furthermore, may contribute to normative calls for deployment votes by scholars and commentators, which in turn influence political decisions to involve the legislature, whether legally required or not.

While our statistical analysis tested the individual explanatory power of the variables and the strength of their relationships, our sub-cases sought to examine their causal interplay. This is an admittedly exploratory design that requires further validation and study. Future studies could deepen our understanding of the causal weight of combinations of conditions to further test the potency of the conjunctural path we found most prevalent – executive ideology and mission-risk – against alternative paths toward deployment votes.

Moreover, the hypotheses we tested in this article were not comprehensive. One hypothesis that was not outlined or tested relates to multiple votes for a single mission. Specifically, it may be that votes to extend existing missions are motivated by different factors, such as the expectation that if the legislature voted on a mission once, it should do so again. Conversely, it may be that a mission that has been voted on once may not be seen to require additional votes. The particularities associated with extension votes or multiple votes for deployments to a single theater should be explored in future research. We also did not test whether the likelihood that a government would lose a vote was a factor in their decision to hold one or not. Strong^111^ observed that vote may be more likely to take place when the executive believes that it has a chance of carrying it. This hypothesis will require further testing. Still another hypothesis that we did not include is the influence of individual leaders. As Strong^112^ argued with respect to the British case, the attitudes of Cameron and May had an impact on their decision to hold deployment votes. A case could be made that Canada’s deployment votes were driven by the attitudes of the Prime Minister, with Mulroney and Harper having a personal commitment to the practice that was not present in Chrétien or Martin, and that appears ambivalent for J. Trudeau. Future research on other cases could test the importance of the attitudes of heads of government in this regard. Finally, Strong^113^ examines how the attitude of members of Parliament affected decisions to hold votes in the United Kingdom. While this factor has an impact in the United Kingdom, given notable backbench independence and influence, it would not apply to the Canadian case or many other democracies with strict party discipline and centralized party control.^114^ Nonetheless, future research should investigate whether the views of individual parliamentarians shape the legislature’s involvement in military deployment decisions.

## Appendix

**Table:**

Mission (year)	Vote
Korean War (1950)	No
Indochina (1954)	No
UN Emergency Force I (1956)	Yes
UN Observation Group in Lebanon (1958)	No
Organisation des Nations unies au Congo (1960)	Yes
UN Peacekeeping Force in Cyprus (1964)	Yes
UN India-Pakistan Observer Mission (1965)	No
UN Emergency Force II (1973)	Yes
Vietnam (1973)	No
UN Disengagement Observer Force (1974)	No
UN Interim Force in Lebanon (1978)	No
Middle East observation mission (1986)	No
UN Iran-Iraq Military Observer Group (1988)	Yes
United Nations Observer Group in Central America (1989)	No
United Nations Transition Assistance Group Namibia (1989)	No
United Nations Observer Mission in El Salvador (1991)	No
Persian Gulf War (1991)	Yes
UN Protection Force in the Balkans (1992)	No
Somalia UNOSOM (1992)	No
Somalia United Task Force (1992)	Yes
United Nations Transitional Authority in Cambodia (1992)	No
United Nations Assistance Mission for Rwanda (1993)	No
United Nations Mission in Haiti (1993)	No
Humanitarian aid mission in Rwanda (1994)	No
NATO Implementation Force in the former Yugoslavia (1995)	No
African Great Lakes Multinational Force (1996)	No
United Nations Support Mission in Haiti (1996)	No
NATO Stabilization Force (1996)	No
Canadian Air Contingent Bosnia-Herzegovina (1997)	No
United Nations Transition Mission in Haiti (1997)	No
Joint Task Force Central America (1998)	No
Joint Task Force Serdivan (1999)	No
International Force in East Timor (1999)	No
NATO Kosovo Force (1999)	No
United Nations Mission in Ethiopia and Eritrea (2000)	No
NATO Operation Essential Harvest (2001)	No
Campaign Against Terrorism in Afghanistan and the Arabian Sea (2001)	No
Operation Apollo in Afghanistan (2002)	No
International Security Assistance Force in Afghanistan (2003)	No
United Nations Multinational Interim Force in Haiti (2004)	No
UN Stabilization Mission in Haiti (2004)	No
Operation Archer in Kandahar, Afghanistan (2005)	No
Kandahar 2006	Yes
Kandahar 2008	Yes
Kabul 2010	No
Libya March 2011	Yes
Libya June 2011	Yes
Libya September 2011	Yes
Central and Eastern Europe 2014	No
ISIL August 2014	No
ISIL October 2014	Yes
ISIL March 2015	Yes
Ukraine 2015	No
ISIL 2016	Yes
Ukraine extension 2017	No
NATO battlegroup in Latvia June 2017	No
NATO battlegroup in Latvia extension July 2018	No
Mali 2018	No
Ukraine extension 2019	No

## Notes

1. Rosara Joseph, *The War Prerogative: History, Reform, and Constitutional Design* (Oxford: Oxford University Press, 2013); Veronika Fikfak and Hayley Hooper, *Parliament’s Secret War* (London: Hart Publishing, 2018); Sarah Burns, *The Politics of War Powers: The Theory and History of Presidential Unilateralism* (Lawrence, KS: University Press of Kansas, 2019); Peter E. Mulherin, ‘Going to War Democratically: Lessons for Australia From Canada and the UK’, *Australian Journal of International Affairs*, 73(4), 2019, pp. 357–75.

2. Heiner Hänggi, ‘The Use of Force Under International Auspices: Parliamentary Accountability and “Democratic Deficits”’, in Hans Born and Heiner Hänggi (eds), *The ‘Double Democratic Deficit’. Parliamentary Accountability and the Use of Force Under International Auspices* (London and New York, NY: Routledge, 2018), pp. 203–15; Ariella Huff, ‘Executive Privilege Reaffirmed? Parliamentary Scrutiny of the CFSP and CSDP’, *West European Politics*, 38(2), 2015, pp. 396–415; James Strong, ‘Interpreting the Syria Vote: Parliament and British Foreign Policy’, *International Affairs*, 91(5), 2015, pp. 1123–39; Julie Kaarbo and Daniel Kenealy, ‘No, Prime Minister: Explaining the House of Commons Vote on Intervention in Syria’, *European Security*, 25(1), 2016, pp. 28–48; Juliet Kaarbo and Daniel Kenealy, ‘Precedents, Parliaments, and Foreign Policy: Historical Analogy in the House of Commons Vote on Syria’, *West European Politics*, 40(1), 2017, pp. 62–79; Patrick A. Mello, ‘Curbing the Royal Prerogative to Use Military Force: The British House of Commons and the Conflicts in Libya and Syria’, *West European Politics*, 40(1), 2017, pp. 80–100; Falk Ostermann, ‘France’s Reluctant Parliamentarisation of Military Deployments. The 2008 Constitutional Reform in Practice’, *West European Politics*, 40(1), 2017, pp. 101–18; Tapio Raunio and Wolfgang Wagner, ‘Towards Parliamentarisation of Foreign and Security Policy?’, *West European Politics*, 40(1), 2017, pp. 1–19; Tapio Raunio and Wolfgang Wagner, ‘Party Politics or (Supra-)National Interest? External Relations Votes in the European Parliament’, *Foreign Policy Analysis*, 16(4), 2020, pp. 547–64; Fabrizio Cottichia and Valerio Vignoli, ‘Italian Political Parties and Military Operations: An Empiral Analysis on Voting Patterns’, *Government and Opposition*, 55(3), 2018, pp. 456–73; Wolfgang Wagner, ‘Is There a Parliamentary Peace? Parliamentary Veto Power and Military Interventions From Kosovo to Daesh’, *British Journal of Politics and International Relations*, 20(1), 2018, pp. 121–34; Wolfgang Wagner, *The Democratic Politics of Military Interventions: Political Parties, Contestation, and Decisions to Use Force Abroad* (Oxford: Oxford University Press, 2020); Fabrizio Cottichia and Francesco N. Moro, ‘Peaceful Legislatures? Parliament and Military Interventions After the Cold War: Insights From Germany and Italy’, *International Relations*, 34(4), 2020, pp. 482–503; Sibel Oktay, *Governing Abroad: Coalition Politics and Foreign Policy in Europe* (Ann Arbor, MI: University of Michigan Press, 2022).

3. Patrick A. Mello, ‘Democratic Peace Theory’, in Paul Joseph (ed.), *The Sage Encyclopedia of War: Social Science Perspectives*, Vol. 1 (Thousand Oaks, CA: SAGE, 2017), pp. 472–476.

4. Roy Rempel, *The Chatter Box: And Insider’s Account of the Irrelevance of Parliament in the Making of Canadian Foreign and Defence Policy* (Toronto, Canada: Dundurn Press, 2002); Norman Hillmer and Philippe Lagassé, ‘Parliament Will Decide: An Interplay of Politics and Principle’, *International Journal*, 71(2), 2016, pp. 328–37; Philippe Lagassé, ‘Parliament and the War Prerogative in the United Kingdom and Canada: Explaining Variations in Institutional Change and Legislative Control’, *Parliamentary Affairs*, 70(2), 2017, pp. 280–300; Jean-Christophe Boucher and Kim R. Nossal, *The Politics of War: Canada’s Afghanistan Mission 2001–2014* (Vancouver: University of British Columbia Press, 2017); Philippe Lagassé and Patrick A. Mello, ‘The Unintended Consequences of Parliamentary Involvement: Elite Collusion and Afghanistan Deployments in Canada and Germany’, *British Journal of Politics and International Relations*, 20(1), 2018, pp. 135–57.

5. Daan Fonck and Yf Reykers, ‘Parliamentarisation as a Two-Way Process: Explaining Prior Parliamentary Consultation for Military Interventions’, *Parliamentary Affairs*, 71(3), 2018, pp. 674–96.

6. Lagassé, ‘Parliament and the War Prerogative in the United Kingdom and Canada’, pp. 280–300.

7. E.g., Joseph, *The War Prerogative: History, Reform, and Constitutional Design*; Fikfak and Hooper, *Parliament’s Secret War*; Burns, *The Politics of War Powers: The Theory and History of Presidential Unilateralism*; Mulherin, ‘Going to War Democratically’, pp. 357–75.

8. Philippe Lagassé, ‘Accountability for National Defence: Ministerial Responsibility, Military Command and Parliamentary Oversight’, *Institute for Research on Public Policy*, No. 4, March 2010, pp. 1–64; Dirk Peters and Wolfgang Wagner, ‘Between Military Efficiency and Democratic Legitimacy: Mapping Parliamentary War Powers in Contemporary Democracies, 1989–2004’, *Parliamentary Affairs*, 64(1), 2011, pp. 175–92.

9. E.g., David P. Auerswald, *Disarmed Democracies: Domestic Institutions and the Use of Force* (Ann Arbor, MI: University of Michigan Press, 2000); Charlotte Ku and Harold K. Jacobson, ‘Broaching the Issues’, in Charlotte Ku and Harold K. Jacobson (eds), *Democratic Accountability and the Use of Force in International Law* (Cambridge: Cambridge University Press, 2003), pp. 3–35; Hänggi, ‘The Use of Force Under International Auspices’; Baris Kesgin and Juliet Kaarbo, ‘When and how Parliaments Influence Foreign Policy: The Case of Turkey’s Iraq Decision’, *International Studies Perspectives*, 11, 2010, pp. 19–36; Patrick A. Mello, *Democratic Participation in Armed Conflict: Military Involvement in Kosovo, Afghanistan, and Iraq* (New York, NY: Palgrave, 2014); Mello, ‘Curbing the Royal Prerogative to Use Military Force’, pp. 80–100; Dirk Peters and Wolfgang Wagner, ‘Executive Privilege or Parliamentary Proviso? Exploring the Sources of Parliamentary War Powers’, *Armed Forces & Society*, 40(2), 2014, pp. 310–31; Sandra Dieterich, Hartwig Hummel and Stefan Marschall, ‘Bringing Democracy Back in: The Democratic Peace, Parliamentary War Powers and European Participation in the 2003 Iraq War’, *Cooperation and Conflict*, 50(1), 2015, pp. 87–106; Huff, ‘Executive Privilege Reaffirmed? Parliamentary Scrutiny of the CFSP and CSDP’, pp. 396–415; James Strong, ‘Interpreting the Syria Vote: Parliament and British Foreign Policy’, *International Affairs*, 91(5), 2015, 1123–39; Tim Haesebrouck, ‘Democratic Participation in the Air Strikes Against Islamic State: A Qualitative Comparative Analysis’, *Foreign Policy Analysis*, 14(2), 2018, pp. 254–75; Kaarbo and Kenealy, ‘No, Prime Minister’, pp. 28–48; Kaarbo and Kenealy, ‘Precedents, Parliaments, and Foreign Policy’, pp. 62–79; Ostermann, ‘France’s Reluctant Parliamentarisation of Military Deployments’, pp. 101–18; Raunio and Wagner, ‘Towards Parliamentarisation of Foreign and Security Policy?’, pp. 1–19; Raunio and Wagner, ‘Party Politics or (Supra-)National Interest?’, pp. 547–64; Cottichia and Vignoli, ‘Italian Political Parties and Military Operations’, pp. 456–73; Wagner, ‘Is There a Parliamentary Peace?’, pp. 121–34; Wagner, *The Democratic Politics of Military Interventions*; Cottichia and Moro, ‘Peaceful Legislatures?’, pp. 482–503; Florian Böller, ‘Vandenberg Vanished: US Congress and the Politicisation of Military Interventions’, *Parliamentary Affairs*, 75(3), 2022, pp. 676–96; Sibel Oktay, *Governing Abroad: Coalition Politics and Foreign Policy in Europe* (Ann Arbor: University of Michigan Press, 2022).

10. Peters and Wagner, ‘Between Military Efficiency and Democratic Legitimacy’, pp. 175–92.

11. Falk Ostermann and Wolfgang Wagner, ‘Introducting the Parliamentary Deployment Vote Dataset’ (Paper prepared for presentation at the Annual Convention of the International Studies Association, online, 5–9 April 2021), p. 4.

12. Ostermann and Wagner, ‘Introducting the Parliamentary Deployment Vote Dataset’, p. 3.

13. Sandra Dieterich, Hartwig Hummel and Stefan Marschall, ‘Parliamentary War Powers: A Survey of 25 European Parliaments’, Geneva Centre for the Democratic Control of Armed forces, Occasional Paper, No. 21, 2010, p. 91.

14. Dirk Peters, Wolfgang Wagner and Nicole Deitelhoff, ‘Parliaments and European Security Policy: Mapping the Parliamentary Field’, *European Integration Online Papers*, 14(1), 2010, p. 23; Mello, *Democratic Participation in Armed Conflict*, p. 33.

15. Patrick A. Mello and Dirk Peters, ‘Parliaments in Security Policy: Involvement, Politicisation and Influence’, *The British Journal of Politics and International Relations*, 20(1), 2018, pp. 6–7.

16. Dieterich et al., ‘Parliamentary War Powers’, p. 94.

17. Joseph, *The War Prerogative*; Peters and Wagner, ‘Executive Privilege or Parliamentary Proviso?’, pp. 310–31.

18. Notable exceptions include Papua New Guinea and Cyprus, whose parliaments have veto powers.

19. James Strong, ‘Why Parliament now Decides on War: Tracing the Growth of the Parliamentary Prerogative Through Syria, Libya and Iraq’, *British Journal of Politics and International Relations*, 17(4), 2015, pp. 604–22; Lagassé, ‘Parliament and the War Prerogative in the United Kingdom and Canada’, pp. 280–300; Mello, ‘Curbing the Royal Prerogative to Use Military Force’, pp. 80–100; James Strong, ‘Did Theresa may Kill the War Powers Convention? Comparing Parliamentary Debates on UK Intervention in Syria in 2013 and 2018’, *Parliamentary Affairs*, 75(2), 2022, pp. 400–19.

20. E.g., Sibel Oktay, ‘Chamber of Opportunities: Legislative Politics and Coalition Security Policy’, *The British Journal of Politics and International Relations*, 20(1), 2018, pp. 104–20.

21. E.g., Yf Reykers and Daan Fonck, ‘Who is Controlling Whom? An Analysis of the Belgian Federal Parliament’s Executive Oversight Capacities Towards the Military Interventions in Libya (2011) and Iraq (2014-2015)’, *Studia Diplomatica*, 68(2), 2015, pp. 91–110; Raunio and Wagner, ‘Towards Parliamentarisation of Foreign and Security Policy?’, pp. 1–19; Wolfgang Wagner, Anna Herranz-Surrallés, Juliet Kaarbo, et al., ‘The Party Politics of Legislative–Executive Relations in Security and Defence Policy’, *West European Politics*, 40(1), 2017, pp. 20–41.

22. Fonck and Reykers, ‘Parliamentarisation as a Two-Way Process’, pp. 674–96.

23. Rempel, *The Chatter Box*; Christopher Dunn, ‘Democracy in the 21st Century: Canada Needs a War Power Act’, *Canadian Parliamentary Review*, 30(3), 2007, pp. 2–3; J. L. Granatstein, *Whose War is it? How Canada can Survive the Post-9/11 World* (Toronto, Canada: CNIB, 2009).

24. Lagassé, ‘Accountability for National Defence’, pp. 1–64; Alexander Bolt, ‘Crown Prerogative Decisions to Deploy the Canadian Forces Internationally, a Fitting Mechanism for a Liberal Democracy’, in D. Michael Jackson and Philippe Lagassé (eds), *Canada and the Crown: Essays on Constitutional Monarchy* (Kingston, Canada: McGill-Queen’s University Press, 2014), pp. 219–236; Alexander Bolt, ‘The “Convention” to Consult Parliament on Decisions to Deploy the Military: A Political Mirage?’, in Michel Bédard and Philippe Lagassé (eds), *The Crown and Parliament* (Montreal, Canada: Éditions Yvon Blais, 2015), pp. 145–72.

25. Lagassé, ‘Parliament and the War Prerogative in the United Kingdom and Canada’, pp. 280–300; Boucher and Nossal, *The Politics of War*; Lagassé and Mello, ‘The Unintended Consequences of Parliamentary Involvement’, pp. 135–57.

26. Wagner et al., ‘The Party Politics of Legislative–Executive Relations in Security and Defence Policy’, pp. 20–41; Wolfgang Wagner, Anna Herranz-Surrallés, Juliet Kaarbo, et al., ‘Party Politics at the Water’s Edge: Contestation of Military Operations in Europe’, *European Political Science Review*, 10(4), 2018, pp. 537–63.

27. Wagner et al., ‘The Party Politics of Legislative–Executive Relations in Security and Defence Policy’, pp. 20–41; Wagner et al., ‘Party Politics at the Water’s Edge’, pp. 537–63.

28. Wagner, *The Democratic Politics of Military Interventions*, p. 146.

29. Wagner et al., ‘The Party Politics of Legislative–Executive Relations in Security and Defence Policy’, p. 25; Wagner, *The Democratic Politics of Military Interventions*, p. 95.

30. Raunio and Wagner, ‘Towards Parliamentarisation of Foreign and Security Policy?’, p. 11.

31. For a wider discussion of parties and partisanship in Canadian foreign and defense policy, see the special edition of *International Journal* edited by Bow and Black (2008–9).

32. Holger Döring, Huber Constantin and Philip Manow, ‘Parliament and Governments Database (ParlGov): Information on Parties, Elections and Cabinets in Modern Democracies’, *Development Version*, n.d. available at: https://www.parlgov.org/data-info/ (accessed 15 September 2021).

33. ParlGov also aggregates data on anti/pro-European Union, but this dimension is of course not coded for Canadian political parties.

34. Ian Budge, Hans-Dieter Klingemann, Andrea Volkens, et al., *Mapping Policy Preferences: Estimates for Parties, Electors, and Governments 1945–1998* (Oxford: Oxford University Press, 2001); Hans-Dieter Klingemann, Andrea Volkens, Judith Bara, et al., ‘*Mapping Policy Preferences II: Estimates for Parties, Electors, and Governments in Eastern Europe, European Union and OECD 1990–2003* (Oxford: Oxford University Press, 2006); Kenneth Benoit and Michael Laver, *Party Policy in Modern Democracies* (New York, NY: Routledge, 2006).

35. William Cross and Lisa Young, ‘Policy Attitudes of Party Members in Canada: Evidence of Ideological Politics’, *Canadian Journal of Political Science*, 35(4), 2002, pp. 859–80.

36. See Christopher Cochrane, ‘Left/Right Ideology and Canadian Politics’, *Canadian Journal of Political Science*, 48(3), 2010, pp. 583–605.

37. Ludovic Rheault and Christopher Cochrane, ‘Word Embeddings for the Analysis of Ideological Placement in Parliamentary Corpora’, *Political Analysis*, 28(1), 2020, pp. 112–33.

38. Tom Flanagan, *Waiting for the Wave: The Reform Party and the Conservative Movement* (Montreal and Kingston: McGill-Queen’s University Press, 2009); Rainer Knopff, ‘Populism and the Politics of Rights: The Dual Attack on Representative Democracy’, *Canadian Journal of Political Science*, 31(4), 1998, pp. 683–705.

39. Hillmer and Lagassé, ‘Parliament Will Decide’, pp. 328–37.

40. David P. Auerswald, ‘Inward Bound: Domestic Institutions and Military Conflicts’, *International Organization*, 53(3), 1999, pp. 469–504.

41. Randolph M. Siverson, ‘Democracies and War Participation: In Defense of the Institutional Constraints Argument’, *European Journal of International Relations*, 1(4), 1995, p. 483.

42. Stefano Recchia, *Reassuring the Reluctant Warriors: U.S. Civil-Military Relations and Multilateral Intervention* (Ithaca, NY: Cornell University Press, 2015).

43. Auerswald, *Disarmed Democracies*; Dan Reiter and Allan C. Stam, *Democracies at War* (Princeton, NJ: Princeton University Press, 2002); Mello, *Democratic Participation in Armed Conflict*; Dieterich et al., ‘Bringing Democracy Back in’, pp. 87–106; Haesebrouck, ‘Democratic Participation in the Air Strikes Against Islamic State’, pp. 254–75; Wagner, ‘Is There a Parliamentary Peace?’, pp. 121–34.

44. Peters and Wagner, ‘Executive Privilege or Parliamentary Proviso?’, p. 321.

45. Ostermann, ‘France’s Reluctant Parliamentarization of Military Deployments’, p. 113; Fonck and Reykers, ‘Parliamentarisation as a Two-Way Process’, p. 680.

46. Fonck and Reykers, ‘Parliamentarisation as a Two-Way Process’, p. 681.

47. Peters et al., ‘Parliaments and European Security Policy’, p. 7.

48. Wagner, ‘Is There a Parliamentary Peace?’, p. 123.

49. Robert D. Putnam, ‘Diplomacy and Domestic Politics: The Logic of Two-Level Games’, *International Organization*, 42(3), 1988, p. 449; James D. Fearon, ‘Domestic Political Audiences and the Escalation of International Disputes’, *The American Political Science Review*, 88(3), 1994, pp. 577–92.

50. Patrick A. Mello, ‘Parliamentary Peace or Partisan Politics? Democracies’ Participation in the Iraq War’, *Journal of International Relations and Development*, 15, 2012, p. 431; Tim Haesebrouck, ‘NATO Burden Sharing in Libya: A Fuzzy Set Qualitative Comparative Analysis’, *Journal of Conflict Resolution*, 61(1), 2017, p. 2244; Tim Haesebrouck and Anouschka van Immerseel, ‘When Does Politics Stop at the Water’s Edge? A QCA of Parliamentary Consensus on Military Deployment Decisions’, *European Political Science Review*, 12(3), 2020, p. 380.

51. Department of National Defence, *Canadian Forces Joint Publication 01. Canadian Military Doctrine* (Ottawa, Canada: Department of National Defence, 2009), pp. 2–13.

52. Department of National Defence, *Canadian Forces Joint Publication 5.1. Canadian Military Doctrine* (Ottawa, Canada: Department of National Defence, 2008).

53. Philippe Lagassé, ‘The Constitutional Politics of Parliament’s Role in International Policy’, in Adam Chapnick and Christopher Kukacha (eds), *The Harper Era in Canadian Foreign Policy: Parliament, Politics, and Canada’s Global Posture* (Vancouver: University of British Columbia Press, 2016), pp. 56–70.

54. E.g., Zeev Maoz and Bruce Russett, ‘Normative and Structural Causes of the Democratic Peace, 1946–1986’, *American Political Science Review*, 87(3), 1993, pp. 624–38; Bruce Bueno de Mesquita and Randolph M. Siverson, ‘War and the Survival of Political Leaders: A Comparative Study of Regime Types and Political Accountability’, *American Political Science Review*, 89(4), 1995, pp. 841–55; George Tsebelis, ‘Decision Making in Political Systems: Veto Players in Presidentialism, Parliamentarism, Multicameralism and Multipartyism’, *British Journal of Political Science*, 25(3), 1995, pp. 239–325; Mello, ‘Democratic Peace Theory’.

55. Dan Reiter and Erik R. Tillman, ‘Public, Legislative, and Executive Constraints on the Democratic Initiation of Conflict’, *Journal of Politics*, 64(3), 2002, p. 814.

56. Dieterich et al., ‘Parliamentary War Powers’, p. 9.

57. Miriam Fendius Elman, ‘Unpacking Democracy: Presidentialism, Parliamentarism, and Theories of Democratic Peace’, *Security Studies*, 9(4), 2000, pp. 91–126.

58. Maoz and Russett, ‘Normative and Structural Causes of the Democratic Peace’, pp. 624–38; Brandon C. Prins and Christopher Sprecher, ‘Institutional Constraints, Political Opposition, and Interstate Dispute Escalation: Evidence From Parliamentary Systems, 1946–89’, *Journal of Peace Research*, 36(3), 1999, pp. 271–87; Auerswald, *Disarmed Democracies*; Michael J. Ireland and Scott Sigmund Gartner, ‘Government Type and Conflict Initiation in Parliamentary Systems’, *Journal of Conflict Resolution*, 45(5), 2001, pp. 547–68; Glenn Palmer, Tamar London and Patrick Regan, ‘What’s Stopping you? The Sources of Political Constraints on International Conflict Behavior in Parliamentary Democracies’, *International Interactions*, 30(1), 2004, pp. 1–24; Stephen M. Saideman and David P. Auerswald, ‘Comparing Caveats: Understanding the Source of National Restrictions Upon NATO’s Mission in Afghanistan’, *International Studies Quarterly*, 56(1), 2012, pp. 67–84; Wagner, ‘Is There a Parliamentary Peace?’, pp. 121–34.

59. In contrast, Brûlé and Williams find that “minority executives appear to have a greater propensity than majority executives to turn to the international arena to demonstrate their leadership competence in response to deteriorating economic conditions.” David J. Brûlé and Laron K. Williams, ‘Democracy and Diversion: Government Arrangements, the Economy, and Dispute Initiation’, *Journal of Peace Research*, 46(6), 2009, p. 687. Reiter and Tillman also failed to find a consistent set of relationships between the type of executive-legislative structure and the propensity to initiate international disputes. Dan Reiter and Erik R. Tillman, ‘Public, Legislative, and Executive Constraints on the Democratic Initiation of Conflict’, *Journal of Politics*, 64(3), 2002, p. 823.

60. R. Kent Weaver, ‘The Politics of Blame Avoidance’, *Journal of Public Policy*, 6(4), 1986, pp. 371–98.

61. Weaver, ‘The Politics of Blame Avoidance’, p. 377.

62. E.g., Christopher Hood, *The Blame Game. Spin, Bureaucracy, and Self-Preservation in Government* (Princeton, NJ and Oxford: Princeton University Press, 2010); R. Kent Weaver, *Policy Leadership and the Blame Trap: Seven Strategies for Avoiding Policy Stalemate* (Washington, DC: Governance Studies at Brookings Institute, 2013).

63. For a review, see Markus Hinterleitner and Fritz Sager, ‘Anticipatory and Reactive Forms of Blame Avoidance: Of Foxes and Lions’, *European Political Science Review*, 9(4), 2017, pp. 587–606.

64. James M. Lindsay, ‘Congress, Foreign Policy, and the New Institutionalism’, *International Studies Quarterly*, 38(2), 1994, pp. 281–304.

65. Lagassé, ‘The Constitutional Politics of Parliament’s Role in International Policy’; Lagassé, ‘Parliament and the War Prerogative in the United Kingdom and Canada’.

66. Fonck and Reykers, ‘Parliamentarisation as a Two-Way Process’, pp. 674–96.

67. Sarah Kreps, ‘Elite Consensus as a Determinant of Alliance Cohesion: Why Public Opinion Hardly Matters for NATO-led Operations in Afghanistan’, *Foreign Policy Analysis*, 6, 2010, pp. 191–215.

68. Lagassé, ‘Accountability for National Defence’, p. 12.

69. Lagassé and Mello, ‘The Unintended Consequences of Parliamentary Involvement’, pp. 135–57.

70. Justin Massie, ‘Public Contestation and Policy Resistance: Canada’s Oversized Military Commitment to Afghanistan’, *Foreign Policy Analysis*, 12(1), 2016, p. 52.

71. Wagner, *The Democratic Politics of Military Interventions*, p. 88.

72. John E. Mueller, *War, Presidents, and Public Opinion* (New York, NY: Wiley, 1973); Brad Lian and John R. Oneal, ‘Presidents, the Use of Military Force, and Public Opinion’, *Journal of Conflict Resolution*, 37(2), 1993, pp. 277–300.

73. Michael Dewing and Corinne McDonald, *International Deployment of Canadian Forces: Parliament’s Role* (Ottawa, Canada: Parliamentary Information and Research Service, 2006).

74. The vast majority of Canadian casualties during the war in Afghanistan occurred between 2006 and 2010. Jean-Christophe Boucher and Kim R. Nossal, *The Politics of War: Canada’s Afghanistan Mission 2001–2014* (Vancouver: University of British Columbia Press, 2017), p. 183.

75. This factor was highlighted in correspondence between the authors and former ministers and senior advisors in the J. Trudeau government.

76. Rempel, *The Chatter Box*, p. 147.

77. Kim Richard Nossal, ‘Quantum Leaping: The Gulf Debate in Australia and Canada’, in Michael McKinley (ed.), *The Gulf War: Critical Perspectives* (Sydney: Allen and Unwin, 1994), pp. 48–71; Kim Richard Nossal, ‘Opening up the Policy Process: Does Party Make a Difference?’, in Nelson Michaud and Kim Richard Nossal (eds), *Diplomatic Departures: The Conservative Era in Canadian Foreign Policy, 1984–93* (Vancouver: University of British Columbia Press, 2001), pp. 276–289.

78. Brian Mulroney, *Memoirs* (Toronto, Canada: McClelland and Stewart, 2007).

79. Douglas Bland and Roy Rempel, ‘A Vigilant Parliament. Building Competence for Effective Parliamentary Oversight of National Defence and the Canadian Armed Forces’, *IRPP Policy Matters*, 5(1), 2004, p. 12.

80. Robert Davis, ‘Canada and the Persian Gulf War’ (Electronic Theses and Dissertations 1354, University of Windsor, Windsor, Canada, 1997), p. 36.

81. Davis, *Canada and the Persian Gulf War*, p. 115.

82. Anthony Wilson-Smith and Bruce Wallace, ‘A Call to Arms: Canadians Debate their Battle Role’, *Maclean’s*, 100(4), 28 January 1991, available at: https://www.macleans.ca/culture/books/a-call-to-arms-for-the-call-of-the-wild (accessed 30 March 2022).

83. Nancy Wood, ‘The Isolationist Province’, *Maclean’s*, 100(4), 28 January 1991, p. 35.

84. John Kirton, ‘National Mythology and Media Coverage: Mobilizing Consent for Canada’s War in the Gulf’, *Political Communication*, 10(4), 1993, pp. 429–31.

85. Nossal, ‘Quantum Leaping’, p. 61.

86. Gregory Wirick, ‘Canada, Peacekeeping and the United Nations’, in Fen Osler Hampson and Christopher J. Maule (eds), *Canada Among Nations 1992-93: A New World Order?* (Ottawa, Canada: Carleton University Press, 1992), pp. 94–114.

87. Four Liberals, three Bloc Québécois MPs and an independent voted against the motion.

88. Davis, *Canada and the Persian Gulf War*, p. 114.

89. Dewing and McDonald, *International Deployment of Canadian Forces*.

90. Janice Stein and Eugene Lang, *The Unexpected War: Canada in Kandahar* (Toronto, Canada: Penguin Canada, 2007).

91. Rempel, *The Chatter Box*.

92. Lagassé, ‘Parliament and the War Prerogative in the United Kingdom and Canada’, p. 291.

93. Conservative Party of Canada (CPC), *Stand up for Canada —Federal Election Platform*, 2006, p. 45.

94. Massie, ‘Public Contestation and Policy Resistance’, p. 59.

95. Ian Brodie, *At the Centre of Government: The Prime Minister and the Limits on Political Power* (Montreal, Canada: McGill-Queen’s University Press, 2018), p. 48.

96. Massie, ‘Public Contestation and Policy Resistance’, pp. 57–8; Boucher and Nossal, *The Politics of War*, p. 157.

97. Brodie, *At the Centre of Government*, p. 49.

98. Brodie, *At the Centre of Government*, pp. 45–51.

99. Boucher and Nossal, *The Politics of War*, pp. 100–16.

100. Justin Massie, ‘Stephen Harper’s War in Afghanistan: Eagerly in, Cautiously out’, in Peter McKenna (ed.), *Harper’s World: The Politicization of Canadian Foreign Policy, 2006–2015* (Toronto, Canada: University of Toronto Press, 2022), p. 161.

101. Harper’s former chief of staff, Ian Brodie (2018), discusses the 2006 and 2008 extensions, but overlooks the 2010 deployment decision. Brodie, *At the Centre of Government*.

102. The combat/non-combat nature of the mission generated some political debate, but military authorities were adamant that ROE authorized the use of force for self-defense purposes only. David Pugliese, ‘Another Name for a Combat Mission’, *Ottawa Citizen*, 9 February 2016, N6; Aron Wherry, ‘Iraq: When Does the “Constructive Dialogue” Start?’, *Maclean’s*, 29 January 2016, available at: https://www.macleans.ca/politics/on-iraq-questions-of-combat-cost-and-clarity/ (accessed 30 March 2022).

103. Justin Massie and Marco Munier, ‘Being a Reliable Ally in a Politicized War: Canada’s Fight Against ISIS’, in Thomas Juneau and Bessma Momani (eds), *Middle Power in the Middle East: Canada’s Foreign and Defence Policies in a Changing Region* (Toronto, Canada: University of Toronto Press, 2022), p. 39.

104. Neil Moss, ‘Canada Enters Dangerous Year-Long Peacekeeping in Mali, Conservatives Still Want House Debate and Vote’, *Hill Times*, 8 August 2018.

105. Laura Pelletier and Justin Massie, ‘Role Conflict: Canada’s Withdrawal From Combat Operations Against ISIL’, *International Journal*, 723, 2017, pp. 298–317.

106. Correspondence between the authors and former ministers and senior advisors in the J. Trudeau government (9 February 2022).

107. Wagner, *The Democratic Politics of Military Interventions*.

108. Strong, ‘Did Theresa may Kill the War Powers Convention?’, pp. 400–19.

109. Mulherin, ‘Going to War Democratically’, pp. 357–75.

110. Reiter and Stam, *Democracies at War*; Mello, *Democratic Participation in Armed Conflict*; Dieterich et al., ‘Bringing Democracy Back in’, pp. 87–106; Haesebrouck, ‘Democratic Participation in the Air Strikes Against Islamic State’, pp. 254–75; Wagner, ‘Is There a Parliamentary Peace?’, pp. 121–34.

111. James Strong, ‘The War Powers of the British Parliament: What has Been Established, and What Remains Unclear?’, *British Journal of Politics and International Relations*, 20(1), 2018, pp. 19–34.

112. Strong, ‘Did Theresa may Kill the War Powers Convention?’, pp. 400–19.

113. Strong, ‘Did Theresa may Kill the War Powers Convention?’, pp. 400–19.

114. Alex Marland, *Whipped: Party Discipline in Canada* (Vancouver: University of British Columbia Press, 2020).

